# Chemotherapy Liberates a Broadening Repertoire of Tumor Antigens for TLR7/8/9-Mediated Potent Antitumor Immunity

**DOI:** 10.3390/cancers17193277

**Published:** 2025-10-09

**Authors:** Cheng Zu, Yiwei Zhong, Shuting Wu, Bin Wang

**Affiliations:** 1 Shanghai Institute of Infectious Disease and Biosecurity, Fudan University, Shanghai 200032, China; zucheng@fudanpci.org (C.Z.); yiweizhong@fudan.edu.cn (Y.Z.); 2 Key Laboratory of Medical Molecular Virology (MOE/NHC/CAMS), School of Basic Medical Sciences, Fudan University, Shanghai 200032, China; april.wu@innoventbio.com; 3 Advaccine Biopharmaceuticals Suzhou Co., Ltd., Suzhou 215000, China

**Keywords:** TLR agonists, chemo-immunotherapy, tumor antigen release, DAMPs, TCR repertoire, precision oncology

## Abstract

**Simple Summary:**

Many tumors are “cold”, resisting immunotherapy because immune cells see too few tumor markers. We tested whether chemotherapy releases tumor antigens and whether CR108, an immune stimulant, amplifies responses. In multiple tumor models, chemotherapies—especially platinum—raised tumor antigens, changed the tumor’s immune environment, and, when combined with CR108, led to strong and lasting tumor control. This combination helped T cells recognize tumor antigens more effectively and promoted a diverse, balanced immune response—not just a general “danger” signal. In human breast cancer, higher antigen exposure/processing gene levels predicted better survival. Together, these results show that pairing the right chemotherapy with CR108 can turn “cold” tumors “hot”, offering a practical strategy to improve cancer immunotherapy.

**Abstract:**

Background: Most immunologically “cold” tumors do not respond durably to checkpoint blockade because tumor antigen (TA) release and presentation are insufficient to prime effective T-cell immunity. While prior work demonstrated synergy between cisplatin and a TLR7/8/9 agonist (CR108) in 4T1 tumors, the underlying mechanism—particularly whether chemotherapy functions as a broad antigen-releasing agent enabling TLR-driven immune amplification—remained undefined. Methods: Using murine models of breast (4T1), melanoma (B16-F10), and colorectal cancer (CT26), we tested multiple chemotherapeutic classes combined with CR108. We quantified intratumoral and systemic soluble TAs, antigen presentation and cross-priming by antigen-presenting cells, tumor-infiltrating lymphocytes, and cytokine production by flow cytometry/ICS. T-cell receptor β (TCRβ) repertoire dynamics in tumor-draining lymph nodes were profiled to assess amplitude and breadth. Tumor microenvironment remodeling was analyzed, and public datasets (e.g., TCGA basal-like breast cancer) were interrogated for expression of genes linked to TA generation/processing and peptide loading. Results: Using cisplatin + CR108 in 4T1 as a benchmark, we demonstrate that diverse chemotherapies—especially platinum agents—broadly increase the repertoire of soluble tumor antigens available for immune recognition. Across regimens, chemotherapy combined with CR108 increased T-cell recognition of candidate TAs and enhanced IFN-γ^+^ CD8^+^ responses, with platinum agents producing the largest expansions in soluble TAs. TCRβ sequencing revealed increased clonal amplitude without loss of repertoire breadth, indicating focused yet diverse antitumor T-cell expansion. Notably, therapeutic efficacy was not predicted by canonical damage-associated molecular pattern (DAMP) signatures but instead correlated with antigen availability and processing capacity. In human basal-like breast cancer, higher expression of genes involved in TA generation and antigen processing/presentation correlated with improved survival. Conclusions: Our findings establish an antigen-centric mechanism underlying chemo–TLR agonist synergy: chemotherapy liberates a broadened repertoire of tumor antigens, which CR108 then leverages via innate immune activation to drive potent, T-cell-mediated antitumor immunity. This framework for rational selection of chemotherapy partners for TLR7/8/9 agonism and support clinical evaluation to convert “cold” tumors into immunologically responsive disease.

## 1. Introduction

Despite remarkable advances in cancer immunotherapy, 70–80% of patients fail to respond to current immune checkpoint inhibitors, particularly those with traditionally immunologically “cold” tumors such as pancreatic, ovarian, and triple-negative breast cancers [1,2,3,4]. This therapeutic resistance stems largely from inadequate tumor antigen presentation and insufficient danger signaling necessary to initiate robust antitumor immunity. While chemotherapy has long been recognized for its immunomodulatory potential, the field lacks a rational framework for matching specific chemotherapeutic agents with immunomodulators based on tumor-specific molecular characteristics—a critical barrier to precision chemo–immunotherapy [5,6]. Effective antitumor immunity depends on the availability and presentation of tumor antigens [7,8,9,10]. Beyond direct cytotoxicity, chemotherapy can reshape this prerequisite by exporting intracellular antigens to the extracellular space, where they become accessible to professional antigen-presenting cells (APCs) [9,11]. Such antigenic cargo—comprising soluble proteins and peptides, protein complexes, and vesicle associated materials—broadens the repertoire of targets that can be captured, processed, and cross-presented to T cells [12,13,14,15,16]. While chemotherapy has long been recognized for its immunomodulatory potential through immunogenic cell death, the precise mechanism by which diverse chemotherapeutic agents synergize with TLR agonists remains undefined.

Different cytotoxic agents engage distinct damage programs (e.g., DNA cross-linking by platinum compounds, nucleotide depletion by antimetabolites, alkylation by temozolomide) [17,18,19]. These programs generate non-identical extracellular cargo, yet they may converge on a shared functional outcome: increased availability of soluble tumor antigens that can be captured, processed, and cross-presented [20,21,22]. While immunogenic cell death and upstream danger signaling have been emphasized, accumulating evidence and our data here indicate that the quantity, routing (export), and processing/loading of released antigens are the primary correlates of durable, antigen-specific responses, providing a rationale for pairing chemotherapy with TLR agonism [19,22,23,24]. Although canonical ICD/DAMP signals are readily observed, cross-regimen and cross-lineage differences in therapeutic benefit correlate weakly with DAMP levels, suggesting that DAMPs act as a permissive context rather than primary drivers under our conditions.

What remains underdefined is a systematic, cross-class and cross-lineage view of chemotherapy-induced antigen release and its immunologic consequences [22,25]. Specifically, we lack direct evidence that mechanistically diverse regimens, despite producing different forms of damage, consistently broaden the extracellular tumor antigen/neoantigen landscape [26] and that this broadened landscape can be functionally read by the immune system [27,28].

Motivated by this antigen-centric view, our prior work compared the combination of CR108 with cisplatin delivered locally either at a conventional (higher) dose or as a microdose via subcutaneous/peritumoral injection [29]. In both settings, the cisplatin + CR108 combo treatment outperformed CR108 alone, whereas cisplatin monotherapy exerted limited benefit. The near dose independence of this improvement suggested that even minimal local cisplatin exposure can unmask tumor antigens in situ, thereby seeding cross-priming that CR108 subsequently amplifies. This observation led us to hypothesize that chemotherapy’s principal contribution to synergy with CR108 is the provision of extracellular TAs and their efficient routing/processing, which CR108 then amplifies through dendritic-cell activation and cytokine programs [30].

Here, we tested this hypothesis across multiple chemotherapy classes and tumor lineages. Using breast (4T1), melanoma (B16-F10), and colorectal (CT26) models, we mapped chemotherapy-unleashed TA cargo by secretome proteomics. To avoid ambiguity, we collectively refer to tumor-derived antigenic species—including TAAs, TSAs/neoantigens, and therapy-exposed self-epitopes—as tumor antigens (TAs). Secondly, we tested functional recognition with peptide-directed IFN-γ ELISPOT and intracellular cytokine staining, thirdly, we performed profiled tumor immune microenvironment (TIME) remodeling by flow cytometry and histology, and finally, we conducted bulk TCRβ sequencing to evaluate clonotype dynamics and repertoire architecture in tumor-draining lymph nodes. Across agents, we asked whether a common denominator—expanded antigen availability—explains the observed synergy with TLR7/8/9 agonism and outlines principles for rational chemo–adjuvant pairing that balances response amplitude with appropriately moderated T-cell receptor breadth without sacrificing efficacy.

## 2. Materials and Methods

### 2.1. Reagents, Cell Lines, and Culture Conditions

Murine 4T1 breast carcinoma cells and CT26 colon carcinoma cells (both from the Cell Bank of the Chinese Academy of Sciences, Shanghai, China) and B16-F10 melanoma cells (gift from the Zhao Chao laboratory, Fudan University, Shanghai, China) were authenticated by short tandem repeat profiling and confirmed mycoplasma-free. Cells were cultured in RPMI-1640 containing 10% (*v*/*v*) fetal bovine serum at 37 °C in 5% CO_2_.

### 2.2. Mice, Husbandry, and Subcutaneous Tumor Implantation

Female BALB/c or C57BL/6 mice (6–8 weeks, specific-pathogen-free; Vital River, Beijing, China) were maintained under SPF conditions with food and water ad libitum. For subcutaneous tumor models, 5 × 10^5^ 4T1 (BALB/c), 5 × 10^5^ CT26 (BALB/c) or 3 × 10^5^ B16-F10 (C57BL/6) cells in 100 µL sterile PBS were injected subcutaneously into the right dorsal flank (back) using a 27-gauge needle (injection time ~5–10 s). Tumors were measured every 2–3 days with digital calipers: volume = 0.52 × L × W^2^ (L, longest dimension; W, perpendicular width). Mice were euthanized when tumor volume reached ≥ 2000 mm^3^ or any single dimension was ≥15 mm, or if humane endpoints were met. Animals were randomized to treatment groups when tumors became palpable (typically day 5–7). Unless stated otherwise, investigators performing caliper measurements and tissue processing were blinded to group allocation. In vivo dosing is expressed as mg/kg, with route, frequency, vehicle composition, and dosing volume specified.

### 2.3. Chemotherapy and CR108 Regimens

When palpable tumors had formed (day 7–9 after inoculation), mice were assigned to receive either local or systemic chemotherapy: Local arm—one peritumoral injection of cisplatin (2 μg kg^−1^; MCE, New York, NY, USA, HY-17394, dissolved in normal saline). Systemic arm—a single tail-vein injection of one of four chemotherapeutics (oxaliplatin 3 mg kg^−1^, gemcitabine 20 mg kg^−1^, temozolomide 10 mg kg^−1^, or pemetrexed 50 mg kg^−1^; all from MCE). Two days later, both arms received a peritumoral subcutaneous dose of the CR108 (50 μg mouse^−1^ cGMP-grade; Suzhou Advaccine Biopharmaceuticals Co., Ltd., Suzhou, China). Each chemotherapeutic agent was tested only in combination with CR108. In every regimen, CR108 was given once every 7 days for three cycles (Q7d × 3), and the formulation was freshly prepared in PBS (pH 7.4) immediately before use.

### 2.4. Secretome Proteomics

4T1 cells (~90% confluence) were washed twice with PBS and incubated 4 h in serum-free, phenol-red-free RPMI-1640 with 0, 40, 80 or 120 µM cisplatin, and then washed and cultured 12 h in fresh serum-free medium (Dalian Meilun Biotechnology Co., Ltd., Dalian, China). All in vitro experiments report final concentrations (µM), with DMSO ≤0.1% unless stated. Conditioned medium was processed as follows to obtain the exosome-depleted soluble fraction: low-speed clarification (300× *g*, 10 min) → cell-debris removal (2000× *g*, 10 min) → optional medium-speed spin (10,000× *g*, 30 min) → ultracentrifugation (100,000× *g*, 90 min) to pellet small extracellular vesicles (including exosomes). The post-100,000× *g* supernatant (exosome-depleted CM) was retained, concentrated with 10 kDa MWCO filters (Millipore, Burlington, VT, USA) to ~20 µL, quantified by BCA, and adjusted to 30 µg total protein in 50 mM ammonium bicarbonate (50 µL) for digestion. Proteins were reduced (TCEP, Sigma, Burlington, VT, USA, 500 mM, 1 µL), alkylated (CAA, Sigma, 500 mM, 3 µL, 30 min, 22 °C), and digested with trypsin (1:50, 16 h, 37 °C). Peptides were acidified (0.1% TFA, Sigma), desalted (MonoSpin C18), and analyzed on a Bruker timsTOF Pro (Bruker, Billerica, MA, USA) with the gradient and PASEF settings as described. Database search used PEAKS ONLINE (v1.4.2020-10-02_113407) against UniProtKB/Swiss-Prot Mus musculus (downloaded on 1 January 2024); precursor tolerance 20 ppm; fragment 0.05 Da; ≤2 missed cleavages; peptide length 6–45 aa; fixed carbamidomethyl-Cys (+57.021 Da); variable met-oxidation (+15.995 Da) and N-terminal acetylation (+42.011 Da); FDR < 1%. For clarity, throughout the manuscript, “soluble antigens” refer to proteins/peptides detected in this exosome-depleted CM supernatant, unless otherwise specified.

### 2.5. Synthetic Peptides and IFN-γ ELISPOT

Candidate MHC class I epitopes were predicted with IEDB (Recommended/NetMHCpan models) using BALB/c (H-2d) alleles H-2-Kd, H-2-Dd, and H-2-Ld and peptide lengths of 8–11 amino acids from the following proteins: Brap, Vimentin, Septin11, Rad23b, Naa25, Spata5, Rpl13a, Tpx2, Birc6, and Smarca4. For each protein, peptides were ranked by combined binding percentile/affinity and IEDB class I immunogenicity scores, and the top three non-redundant peptides were synthesized at ≥95% purity (GenScript, Nanjing, China) and pooled per protein at equal amounts to generate three peptide stimulation pools. Draining-lymph-node cells (2 × 10^6^ per well) were incubated with the corresponding peptide pool (10 μg mL^−1^ total peptide, 22 h, 37 °C, 5% CO_2_), and IFN-γ spots were developed and enumerated on an AID ELISPOT reader (AID Autoimmune Diagnostika GmbH, Strassberg, Germany). Negative (no peptide) and positive (PMA+ Ionomycin) control wells were included to verify assay performance.

### 2.6. Flow Cytometry Phenotyping

Cells were stained with viability dye, Fc-blocked (anti-CD16/32, 10 min, 22 °C) and incubated with surface antibodies (1 µg mL^−1^, 30 min, 4 °C, dark). After two washes, cells were fixed/permeabilized (Foxp3/Transcription Factor buffer, 30–60 min, 4 °C), washed and stained intracellularly (3 µg mL^−1^, 30–60 min, 4 °C). Events (1–3 × 10^5^) were acquired on a BD LSRFortessa (BD Biosciences, San Jose, CA, USA) and analyzed in FlowJo v10 (FlowJo LLC, Ashland, OR, USA).

### 2.7. Immunofluorescence and Immunohistochemistry

Formalin-fixed (4% paraformaldehyde) paraffin sections (5 µm) were baked (60 °C), de-waxed, rehydrated, permeabilized (0.1% Triton X-100, 5–10 min) and retrieved (0.01 M citrate, pH 6.0, microwave, 10–20 min). Sections were incubated with TUNEL mix (TdT, Sangon Biotech Co., Ltd., Shanghai, China, 1 h, 37 °C), counterstained with DAPI (1 µg mL^−1^, 5 min) and mounted. Red TUNEL-positive nuclei were imaged by fluorescence/confocal microscopy.

### 2.8. Sample Collection and T-Cell Isolation

Tumor-draining lymph nodes (dLNs) were collected from 4T1-bearing BALB/c mice assigned to PBS, cisplatin (peritumoral), CR108, or cisplatin + CR108 per the study design. Tissues were harvested 7 days after the final CR108 dose (endpoint window used elsewhere in this study). dLNs were mechanically dissociated on ice through 70 µm strainers, red blood cells were lysed, and single-cell suspensions were processed blinded to group allocation. CD4^+^ and CD8^+^ T cells were isolated in parallel by magnetic negative selection (spot-checked purity ≥90%) and advanced as separate inputs.

### 2.9. Nucleic Acid Preparation

Genomic DNA (primary workflow) was extracted from each CD4^+^ or CD8^+^ fraction to minimize expression-level bias. In occasional confirmatory samples, total RNA was isolated and reverse-transcribed with random hexamers to cDNA before library construction; gDNA and cDNA were never mixed within a library.

### 2.10. Library Construction (Two-Step Multiplex PCR)

Tcrb libraries targeting CDR3-spanning V(D)J regions were generated by a two-step PCR. The first PCR amplified TCRβ with constant, region–anchored, multiplexed V-gene primers while appending partial adapters; the second PCR introduced unique dual indexes and full sequencing adapters (Jia’an Jianda Medical Technology Co., Ltd., Shanghai, China). All samples within a batch used the same reagent lots and cycling program. Amplicons were bead-purified, quantified, assessed by fragment analysis to confirm the expected size distribution, and pooled equimolarly.

### 2.11. Sequencing Platform and Run Conditions

Pooled libraries were sequenced on a DNBSEQ-T7 instrument (MGI Tech Co., Ltd., Shenzhen, China), paired-end 150 bp, to a depth sufficient for clonotype saturation per platform recommendations. Raw data were delivered as FASTQ files following standard demultiplexing.

### 2.12. Primary Read Processing

Adapters and low-quality bases were trimmed prior to alignment. Reads were processed with MiXCR (v3.0.12) using IMGT germline references for V/D/J assignment, CDR3 extraction, and built-in error correction and clone collapsing.

### 2.13. Clonotype Definition and Productive Filters

Only productive rearrangements were retained (in-frame, no stop codon, valid CDR3 per IMGT). Clonotypes were defined as unique combinations of CDR3 amino-acid sequence with corresponding TRBV and TRBJ calls. All reads mapping to the same clonotype were aggregated as clonotype counts for downstream repertoire summaries.

### 2.14. Quality Control and Inclusion Criteria

Libraries were inspected for complexity, expected insert size, and index integrity. Reads with index collisions/mismatches or signs of index hopping were removed; unique dual-index pairing was enforced. Samples failing minimum input, total reads, or productive-read thresholds were excluded a priori. Negative (no-template) controls were processed alongside experimental libraries to monitor contamination.

### 2.15. Repertoire Summaries for Figure Generation

From productive clonotypes, we derived (i) CDR3 length distributions at the amino-acid level, (ii) TRBV–TRBJ pairing matrices (normalized within sample by total productive reads), and (iii) qualitative treatment-emergent clonotypes, defined for visualization as clonotypes are present in a treatment group but absent in contemporaneously processed PBS controls after down-sampling each comparison to the smaller productive-read count. Uniform pipelines and normalization were applied across groups to permit direct qualitative comparison of repertoire architecture.

### 2.16. cBioPortal Analysis

We queried cBioPortal (https://www.cbioportal.org, accessed on 25 August 2025) for the TCGA Breast Invasive Carcinoma PanCancer Atlas cohort and restricted samples to thePAM50 Basal-like subtype. Overall survival (OS, months) and vital status were extracted from the clinical table and cases with missing/invalid OS were excluded, yielding a final analytic set of *N* = 169 (low-risk *n* = 147; high-risk *n* = 22). Prognostic groups were defined by the cohort’s Kaplan–Meier median OS: events occurring before the median were labeled high-risk, otherwise they were labeled low-risk. mRNA expressions for GPLD1, CHMP6, SNX27, and WDFY3 were obtained as RNA-seq RSEM values (log2 normalized z-scores, cBioPortal default). Group comparisons (low- vs. high-risk) were performed using two-sided Wilcoxon rank-sum tests, and results were visualized as per-sample points overlaid with the median and IQR. Analyses were conducted in R (v4.x); the portal version/date, subtype definition, OS rule, and expression metric were held constant for reproducibility.

### 2.17. GEPIA Analysis

Survival analyses were performed in GEPIA/GEPIA2 (http://gepia.cancer-pku.cn, accessed on 25 August 2025) using the BRCA Basal-like subset (TCGA tumor samples only). Two predefined gene sets were evaluated: ER loading/peptide trimming and TAP loading (CALR, CANX, ERAP1, ERAP2, HSP90B1, PDIA3, TAP1, TAP2, TAPBP, TAPBPL) and endosomal–lysosomal cathepsins (CTSB, CTSD, CTSH, CTSS). For each set, GEPIA’s default multi-gene signature was computed as the mean of log_2_(TPM + 1) expression across genes and samples were dichotomized at the median (50%) into High and Low groups (*n*(High) = *n*(Low) = 535). Overall survival (OS) was assessed with Kaplan–Meier curves, reporting hazard ratios (High vs. Low, 95% CI) and log-rank *p* values. Default GEPIA options were otherwise maintained; exported KM plots and parameter snapshots were archived to ensure exact reproducibility.

### 2.18. Statistics

Analyses were performed using GraphPad Prism 9 (GraphPad Software, San Diego, CA, USA). For two groups, a two-sided unpaired *t*-test was performed. For multiple groups/growth curves, a one-/two-way ANOVA with Tukey or Šidák correction was performed. For survival curves, a log-rank test was performed. The significance was defined as *p* < 0.05.

### 2.19. Ethics

Animal studies were approved by the Fudan University (protocol 20241112-004, 12 November 2024) and adhered to ARRIVE guidelines.

## 3. Results

### 3.1. Re-Establishing Cisplatin–CR108 Efficacy in 4T1 as a Benchmark for Mechanistic Analyses

**Figure 1:**
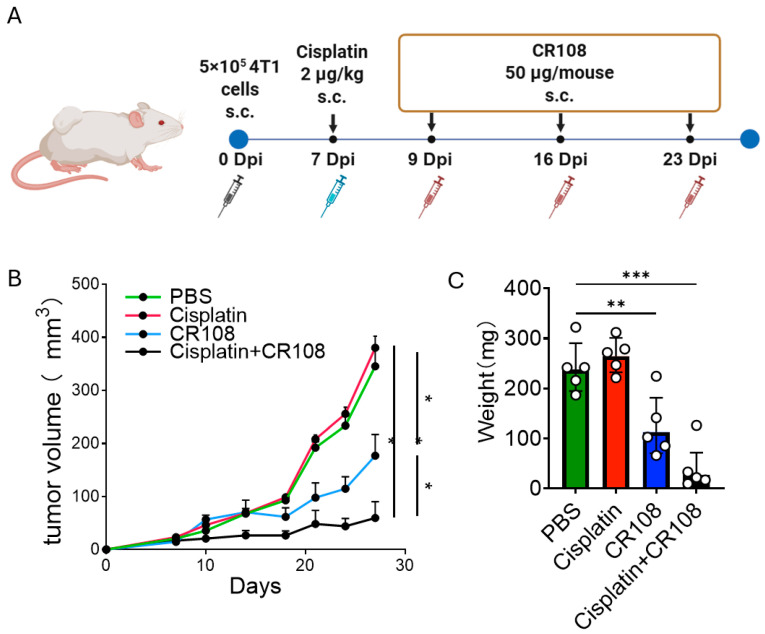
Cisplatin and CR108 cooperate to suppress 4T1 tumor in vivo. (**A**) Treatment schematic. Tumor-bearing mice received low-dose cisplatin (2 µg kg^−1^, peritumoral) on day 0 and CR108 (50 µg, peritumoral) on days 0, 16, and 23. (**B**) Growth curves of subcutaneous 4T1 tumors (mean ± s.e.m.; *n* = 5). Combination therapy produces a rapid inflection of the curve and durable suppression. (**C**) Endpoint 4T1 tumor masses harvested on day 21. Boxes denote median ± inter-quartile range; whiskers, min–max; one-way ANOVA with Tukey post-test (* *p* < 0.05; ** *p* < 0.01; *** *p* < 0.001).

To determine whether cisplatin-induced immunogenic cell death can be amplified in vivo by a Toll-like receptor adjuvant, we first established subcutaneous 4T1 tumors in the right dorsal flank of female BALB/c mice (5 × 10^5^ cells in 100 μL PBS). When tumors became palpable (days 5–7), animals were randomized to receive PBS, low-dose cisplatin, CR108, or the combination (Figure 1A). Low-dose cisplatin alone (2 µg kg^−1^, peritumoral) produced only a modest delay in growth, whereas CR108, administered once weekly for three doses (50 μg, q7d × 3), reduced mean tumor volume by ~50% after two weeks (*p* < 0.05). Consistent with our prior study [29], the combination reproduced early tumor control; here, this experiment serves as a baseline for the downstream mechanistic assays (*p* < 0.001, two-way ANOVA with Tukey correction; *n* = 5). Endpoint masses matched volumes; these data validate the model context for mechanism-focused analyses. (Figure 1B,C)

### 3.2. Cisplatin Triggers Immunogenic Cell Death (ICD) in 4T1 Cells and Promotes the Release of Damage-Associated Molecular Patterns (DAMPs) and Intracellular Cargo

**Figure 2:**
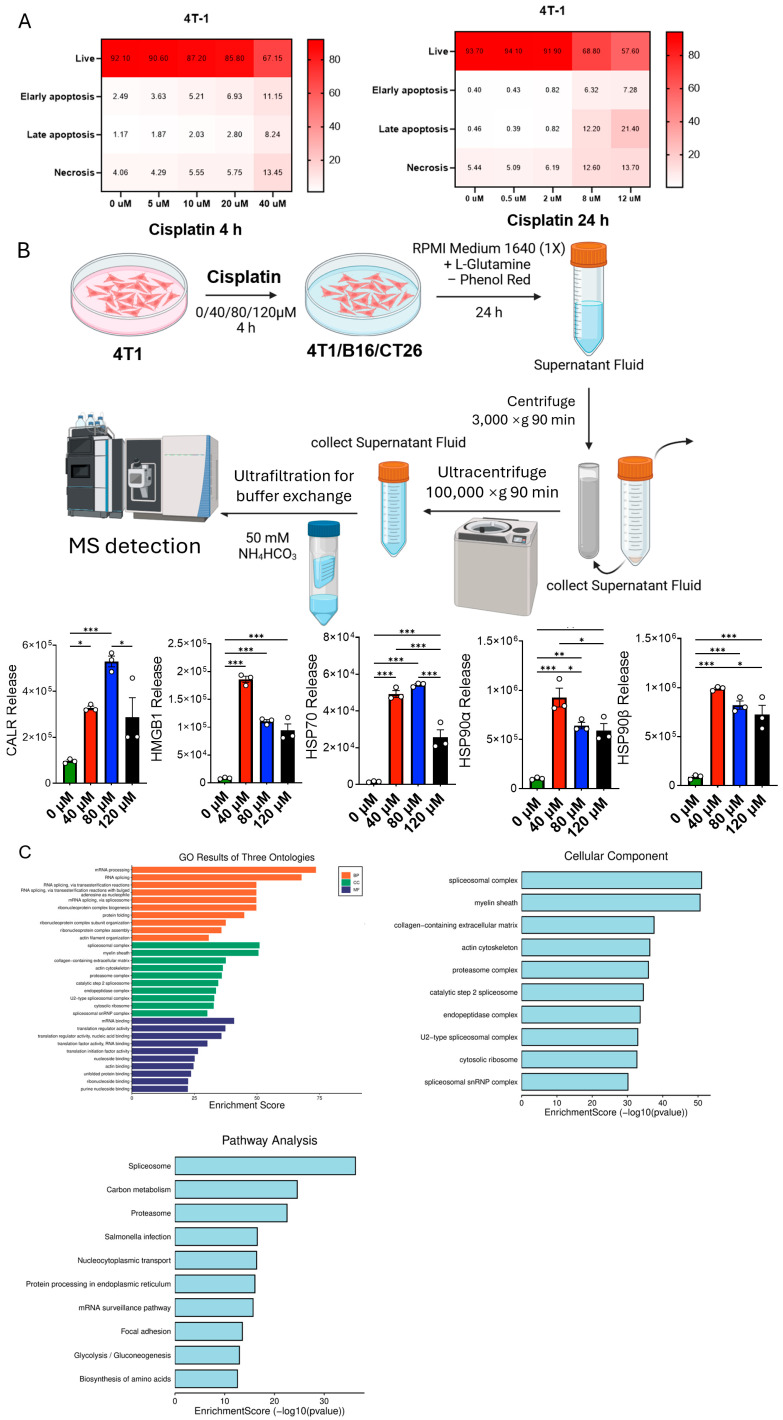
Cisplatin triggers immunogenic cell death (ICD) in 4T1 cells and promotes the release of damage-associated molecular patterns (DAMPs) and intracellular cargo. (**A**) Heat-map of death modalities after 4 h or 24 h exposure to graded cisplatin. 4T1 cells were treated with 0–40 µM cisplatin for 4 h or 0–12 µM for 24 h, stained with Annexin V and propidium iodide, and analyzed by flow cytometry. Row color intensity denotes the percentage of each population (Live, Early apoptosis, Late apoptosis, Necrosis). A 4 h exposure required ≥20 µM cisplatin to provoke appreciable apoptosis or necrosis, whereas 24 h exposure killed cells effectively at 2–8 µM, demonstrating a clear time- and dose-dependent effect. (**B**) Proteomic workflow and quantification of secreted DAMPs. 4T1 cells were exposed to 0, 40, 80 or 120 µM cisplatin for 4 h, washed, incubated for a further 24 h in phenol-red-free medium and the supernatants sequentially cleared (3000× *g*), ultracentrifuged (100,000× *g*) and buffer-exchanged before analysis on a timsTOF mass spectrometer. Increasing cisplatin doses produced corresponding rises in canonical ICD markers CALR, HMGB1, HSP70, and HSP90α/β in the supernatant (*n* = 3; * *p* < 0.05, ** *p* < 0.01, *** *p* < 0.001). (**C**) GO/KEGG enrichment of extracellular proteins identified. Proteins most significantly enriched originate from ribonucleoprotein complexes, endoplasmic-reticulum-associated compartments and the cytoskeleton, consistent with extensive membrane permeabilization and organelle stress during ICD.

As illustrated in Figure 2A, 4T1 mammary carcinoma cells were exposed to a concentration gradient of cisplatin for 4 h (0–40 µM) or 24 h (0–12 µM) and analyzed by Annexin V/propidium-iodide flow cytometry. A 4 h treatment triggered appreciable early or late apoptosis and necrosis only at doses ≥20 µM, whereas extending the exposure to 24 h produced pronounced cell death at 2–8 µM, revealing clear time- and dose-dependent cytotoxicity. Supernatants harvested from cells treated for four hours with 0, 40, 80, or 120 µM cisplatin and incubated for a further 24 h were sequentially clarified (3000× *g*), ultracentrifuged (100,000× *g*), and ultrafiltered before ELISA and timsTOF proteomic analyses. Figure 2B shows that the canonical ICD markers CALR, HMGB1, HSP70, and HSP90α/β increased markedly with rising cisplatin doses, confirming robust DAMP release. Gene Ontology and KEGG annotation of the same proteomic dataset (Figure 2C) indicated significant enrichment of proteins derived from ribonucleoprotein complexes, endoplasmic-reticulum-associated compartments and the cytoskeleton, consistent with extensive membrane permeabilization that discharges intracellular constituents into the tumor microenvironment.

### 3.3. Broadening of the Tumor Antigen (TA) Landscape by Diverse Chemotherapeutics in Three Murine Cancer Models

**Figure 3:**
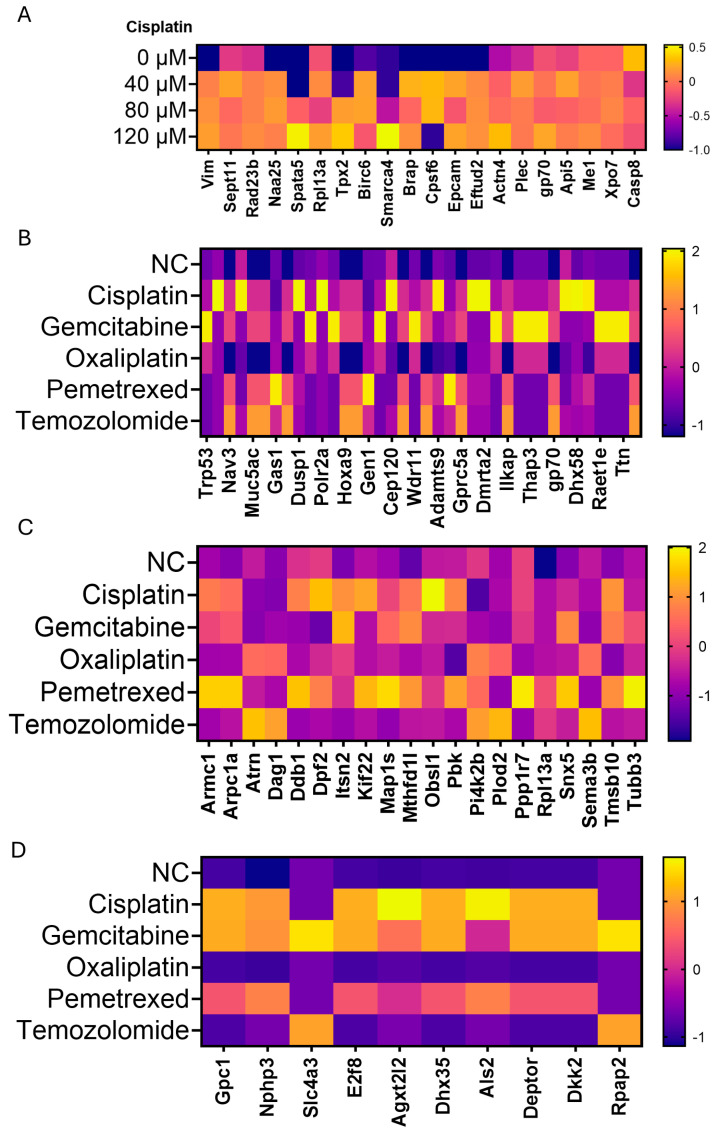
Broadening of the tumor-neoantigen landscape by diverse chemotherapeutics in three murine cancer models. (**A**) Heat-map of candidate neoantigens detected in the supernatant of 4T1 cells treated with cisplatin (0, 40, 80, or 120 µM, 4 h) and incubated for a further 24 h. Color scale (−1.0, purple to 0.5, yellow) denotes the relative change in protein abundance versus untreated control. (**B**) Comparative heat-map of neoantigen-enriched proteins released from 4T1 cells after 4 h exposure to cisplatin, gemcitabine, oxaliplatin, pemetrexed, or temozolomide, quantified by timsTOF mass spectrometry; color scale −1.0 to 1.0. (**C**) Heat-map of putative neoantigens secreted by B16-F10 melanoma cells after 4 h treatment with the same drug panel; color scale −1.0 to 2.0. (**D**) Heat-map of neoantigens released from CT26 colon carcinoma cells under identical conditions; color scale −1.0 to 1.8.

We first curated the targets by surveying the literature to compile, for each cell line, tumor-associated antigens (TAAs), tumor-specific antigens (TSAs) and neoantigens previously reported in that model. Diverse chemotherapeutics broaden the repertoire of soluble TAs across multiple cancer models. In 4T1 cells (Figure 3A), quantitative timsTOF profiling of supernatants collected after cisplatin treatment (0, 40, 80, 120 µM; 4 h followed by a 24 h chase) showed a dose-responsive enrichment of soluble TAs. Alongside established TAs (gp70, EpCAM, vimentin), the analysis revealed elevated candidates—including Sept11, Actn4, Casp8, and Birc6—indicating that cisplatin increases the extracellular availability of soluble tumor-derived epitopes. In head-to-head comparisons (Figure 3B), platinum agents yielded the greatest number and abundance of soluble TAs, whereas antimetabolites and temozolomide preferentially elevated Brap, Tpx2, Rad23b, and related species, revealing mechanism-specific soluble TAs fingerprints. This pattern extended across tumor types: in B16-F10 melanoma (Figure 3C), platinum drugs preferentially enriched DNA-repair/cell-cycle proteins (Ddb1, Kif22, Tubb3, Pbk) among soluble TAs, while gemcitabine/pemetrexed increased nucleotide-metabolism factors; temozolomide elevated Herc2 and Map3k6. In CT26 colon carcinoma (Figure 3D), immunogenic proteins such as Gpc1, E2f8, Agxt2l2, and Deptor rose after treatment. Collectively, these proteomic data indicate that cisplatin dose-dependently increases soluble, tumor-derived TAs in 4T1, that distinct chemotherapeutic classes generate distinct soluble TA signatures within the same tumor.

### 3.4. Cisplatin + CR108 Increase T-Cell Recognition of Candidate TA Peptides in Tumor-Draining LNs

**Figure 4:**
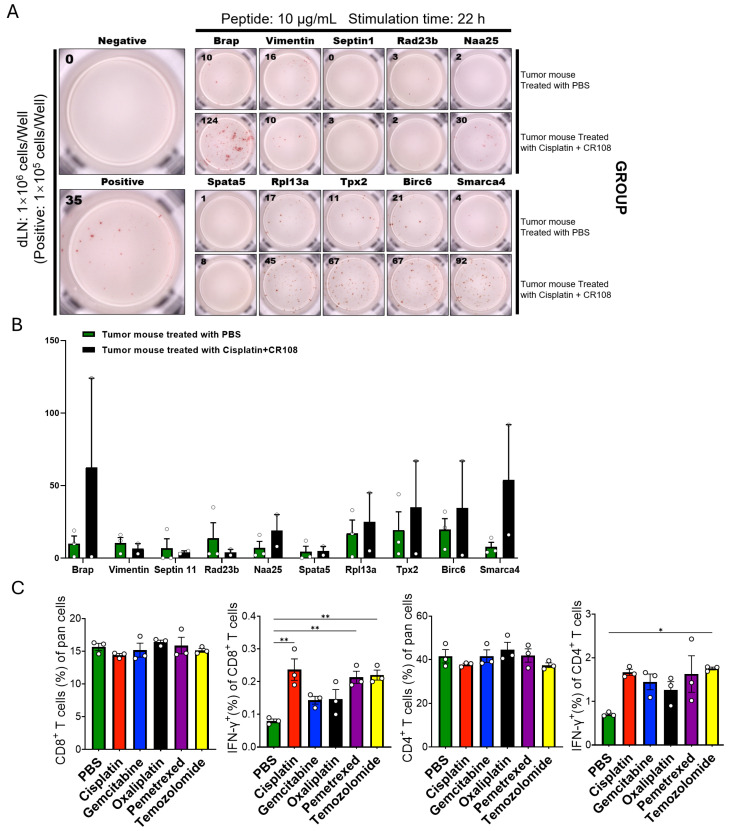
Neoantigen peptides corresponding to proteins identified as enriched in cisplatin-treated secretomes elicit robust T-cell responses—specifically in mice treated with cisplatin + CR108-in tumor- dLNs. (**A**) IFN-γ ELISPOT plate scan. dLN lymphocytes from mice treated with PBS or cisplatin + CR108 were cultured for 22 h in the presence of synthetic peptides corresponding to Brap, Vimentin, Septin11, Rad23b, Naa25, Spata5, Rpl13a, Tpx2, Birc6, and Smarca4 (10 µg mL^−1^ each); individual wells display IFN-γ-secreting spots generated by pooled three-peptide mixture per protein. (**B**) Quantification of IFN-γ spots. Bars represent mean ± s.d.; blue, PBS; green, cisplatin + CR108. The combination group shows significant increases for peptides such as Brap, Rpl13a, and Birc6, whereas PBS wells remain almost blank. (**C**) Flow-cytometric intracellular cytokine staining of dLN T cells. The frequency of IFN-γ^+^ CD8^+^ T cells is markedly higher after cisplatin, gemcitabine, or oxaliplatin treatment than after PBS, and gemcitabine further expands the IFN-γ^+^ CD4^+^ subset. Together, these data confirm that chemotherapy-liberated, CR108-amplified neoantigens are processed and presented in vivo, driving antigen-specific effector T-cell activation(*n* = 3; * *p* < 0.05, ** *p* < 0.01).

We first used the IEDB tools to perform MHC class I binding predictions for the identified proteins (Brap, Vimentin, Septin11, Rad23b, Naa25, Spata5, Rpl13a, Tpx2, Birc6, Smarca4), selecting BALB/c mice (H-2^d^ haplotype) alleles H-2-K^d^, H-2-D^d^, and H-2-L^d and generating 8–11-mer peptides. Based on combined binding affinity/percentile rank and immunogenicity scores, the top three peptides for each protein were synthesized and, for each protein, pooled as a three-peptide mixture for stimulation. Draining lymph node cells from combination-treated (CR108 + cisplatin) or PBS-treated mice were then co-incubated with the pooled peptides (10 μg mL^−1^) for 22 h and subjected to IFN-γ ELISPOT. In the IFN-γ ELISPOT, CR108 + cisplatin dLN cells exhibited higher spot counts than PBS across several candidate TA peptide pools with substantial inter-individual variability (Figure 4A,B). Responses to Brap and Smarca4 ranked highest on average, whereas Tpx2, Birc6, Rpl13a formed an intermediate tier, and Vimentin, Septin11, Spata5, and Rad23b were near background. Consistent trends were observed by intracellular cytokine staining, with increased frequencies of IFN-γ^+^ CD8^+^ (and to a lesser extent CD4^+^) T cells in the combination group (Figure 4C). Because single-agent chemotherapy and single-agent CR108 were not included in these peptide-directed assays, we interpret the data as evidence that under combination conditions, dLN T cells can recognize a subset of candidate TA peptides, rather than attributing the increase to a specific component of the regimen (Figure 4C).

### 3.5. Diverse Chemotherapeutics Synergize with CR108 to Suppress 4T1 and B16-F10 Tumors and Remodel the Immune Microenvironment

**Figure 5:**
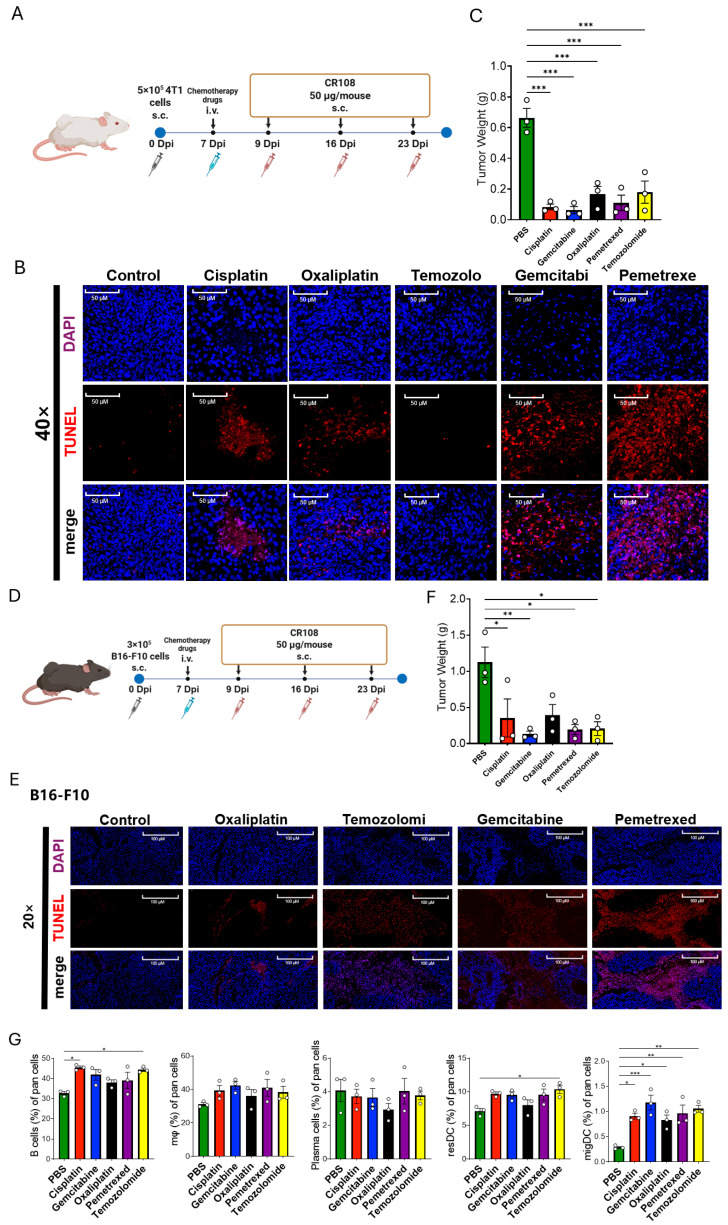
Diverse chemotherapeutics synergize with CR108 to suppress 4T1 and B16-F10 tumors and remodel the immune microenvironment. (**A**) Treatment schedule for 4T1 model. Tumor-bearing BALB/c mice received a single i.v. dose of oxaliplatin (3 mg kg^−1^), temozolomide (10 mg kg^−1^), gemcitabine (20 mg kg^−1^) or pemetrexed (50 mg kg^−1^) on day 7, followed by peritumoral CR108 on days 9, 16, and 23. (**B**) TUNEL/DAPI staining of 4T1 tumors 48 h after chemotherapy. Red, TUNEL; blue, DAPI; merge, bottom row; 40× magnification. All drugs induce extensive apoptosis/necrosis, with gemcitabine and pemetrexed showing the densest signal. (**C**) Endpoint tumor mass for 4T1 (day 30). All combination regimens significantly reduce tumor weight compared with PBS controls. (**D**) Treatment schedule for B16-F10 model. C57BL/6 mice were implanted with 3 × 10^5^ B16-F10 cells (day 0), infused intravenously with cisplatin, oxaliplatin, temozolomide, gemcitabine, or pemetrexed on day 7, and injected with CR108 on days 9, 16 and 23. (**E**) TUNEL/DAPI staining of B16-F10 tumors 48 h after chemotherapy. All drug + CR108 combinations yield broad TUNEL positivity, whereas PBS lesions are largely negative. (**F**) Endpoint tumor mass for B16-F10 (day 30). Each combination markedly inhibits tumor growth (*p* < 0.05 to *p* < 0.001, one-way ANOVA with Tukey post-test). (**G**) Immune-cell composition of 4T1 tumors on day 30. Flow cytometry of single-cell suspensions shows increases in B cells, plasma cells, and migratory dendritic cells (migDCs), the latter most pronounced with gemcitabine and temozolomide. IFN-γ^+^ CD8^+^ T cells rise significantly with cisplatin, gemcitabine, and oxaliplatin, whereas IFN-γ^+^ CD4^+^ T cells increase chiefly after gemcitabine. Data are mean ± s.d. (*n* = 3; * *p* < 0.05, ** *p* < 0.01, *** *p* < 0.001).

Combined treatment with CR108 and mechanistically distinct chemotherapeutic agents produced a robust, model-spanning antitumor synergy while reshaping the immune landscape of 4T1 and B16-F10 tumors. In both models, mice received a single intravenous dose of cisplatin, oxaliplatin, gemcitabine, pemetrexed, or temozolomide on day 7, followed by peritumoral CR108 administrations on days 9, 16, and 23 (Figure 5A,D). Within 48 h of chemotherapy dosing (i.e., prior to the first CR108 injection), TUNEL staining revealed extensive apoptotic and necrotic foci: pemetrexed and gemcitabine generated the most concentrated signal in 4T1 lesions, whereas every drug produced widespread positivity in B16-F10 tumors (Figure 5B,E). By day 30, terminal tumor masses were significantly reduced in all combination groups; oxaliplatin and gemcitabine achieved the greatest shrinkage in 4T1, whereas cisplatin and oxaliplatin were most effective in B16-F10, with pemetrexed (50 mg kg^−1^) and temozolomide (10 mg kg^−1^), nearly eradicating tumors in both settings (Figure 5C,F). Flow-cytometric profiling of 4T1 tumor digests showed a consistent rise in B cells and plasma cells across regimens, a marked expansion of migratory dendritic cells after gemcitabine or temozolomide, sharp increases in IFN-γ^+^ CD8^+^ T cells after cisplatin, gemcitabine and oxaliplatin, and a pronounced enrichment of IFN-γ^+^ CD4^+^ T cells in the gemcitabine cohort (Figure 5G). These data indicate that, irrespective of their primary mode of action, chemotherapeutic agents can, when paired with a TLR-based adjuvant, rapidly induce tumor-cell death, expose antigenic cargo, and mobilize a dendritic-cell-centered, multilineage immune response, thereby achieving durable, mechanism-agnostic tumor control.

### 3.6. Chemotherapeutic Mechanism and Tumor Lineage Shape TAs Profiles; DAMP Fingerprints Vary but Do Not Predict Benefit

**Figure 6:**
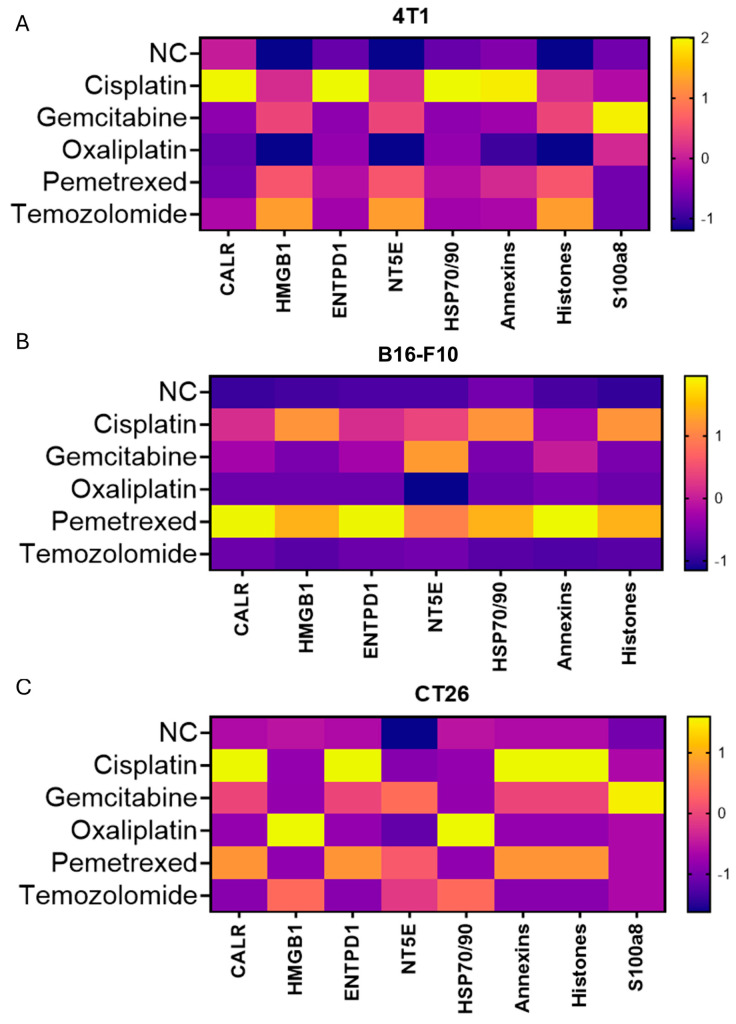
Chemotherapeutic mechanism and tumor lineage shape heterogeneous extracellular cargo/antigen profiles; combination benefit with CR108 is preserved across profiles. (**A**) Heat-map of 7 canonical DAMPs released from 4T1 cells after 4 h treatment with cisplatin, gemcitabine, oxaliplatin, pemetrexed, or temozolomide. Color scale, −1 (purple) to +1 or +2 (yellow), indicates z-score-normalized change relative to untreated control. (**B**) Corresponding DAMP profiles in B16-F10 melanoma cells. Cisplatin and oxaliplatin elicit the strongest CALR translocation and HMGB1/HSP70/90 release, whereas antimetabolites trigger milder yet distinct signatures, highlighting lineage-specific sensitivities. (**C**) DAMP signatures in CT26 colon carcinoma cells. Platinum-driven CALR/HSP peaks persist, but antimetabolite-associated ENTPD1/NT5E induction is accentuated compared with B16-F10, underscoring the impact of tumor genotype on ICD output. Collectively, the heat-maps demonstrate that platinum drugs activate a CALR–HMGB1–HSP axis consistent with DNA cross-link-induced ER stress, antimetabolites promote an ATP–adenosine pathway via ENTPD1/NT5E up-regulation, and temozolomide generates a milder DNA-repair-centered profile. These two variables—drug mechanism and tumor genetics—intersect to generate unique “danger-signal” constellations, offering a molecular rationale for tailoring chemo–TLR–adjuvant pairings and for integrating adenosine- or ER-stress-targeted co-therapies.

Across 4T1, B16-F10, and CT26, chemotherapies generated distinct soluble TA profiles in the exosome-depleted CM (Figure 3), consistent with drug mechanism and lineage. In parallel, a seven-analyte survey of canonical DAMPs revealed class- and lineage-modulated patterns (Figure 6), but no single DAMP or composite score correlated with the magnitude of CR108 synergy across regimens. These observations support an antigen-centric view in which TA availability and routing/processing are the proximate correlates of efficacy, whereas DAMP fingerprints function as a permissive background rather than primary drivers under our conditions.

### 3.7. Synergistic Cisplatin–CR108 Interplay Remodels TCR Abundance While Preserving Repertoire Architecture

**Figure 7:**
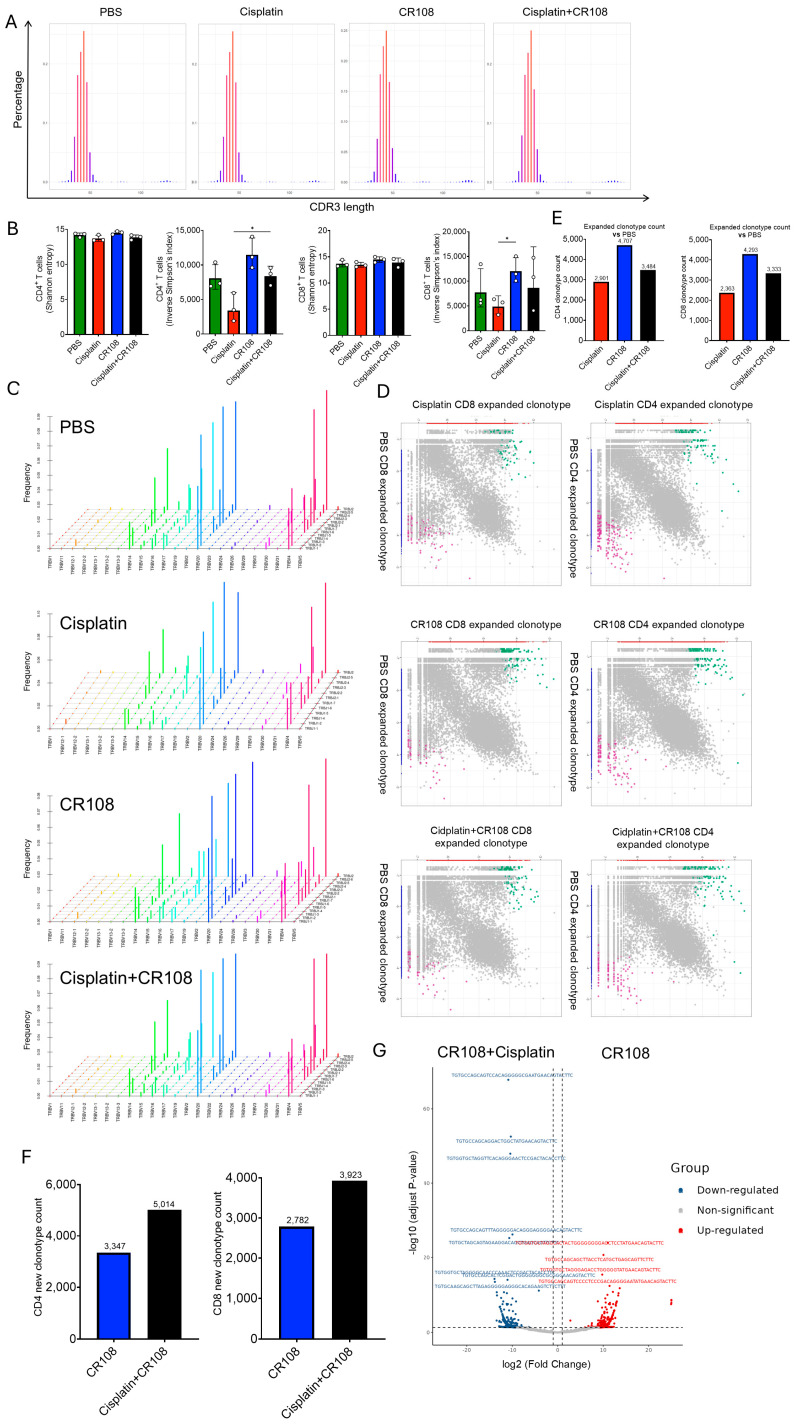
TCR repertoire features in 4T1 tumor-draining lymph nodes under cisplatin–CR108. (**A**) CD8^+^ CDR3 length distributions for PBS, cisplatin, CR108, and combination groups; curves overlap with no significant differences. (**B**) Diversity metrics: cisplatin lowers the Inverse Simpson index in CD8^+^ (*n* = 3; * *p* < 0.05) without affecting Shannon entropy; CR108 increases the Inverse Simpson index in CD4^+^ (*n* = 3; * *p* < 0.05) with a similar tendency in CD8^+^; the combination restores Inverse Simpson to PBS-like levels while yielding the greatest tumor shrinkage. (**C**) TRBV–TRBJ pairing heat-maps for CD8^+^, showing no significant between-group differences, consistent with stable structural features and abundance-level remodeling as the dominant treatment effect. (**D**) Pairwise, clone-wise abundance scatter plots (treatment vs. PBS) for CD4^+^ and CD8^+^ dLN repertoires. Points are colored by differential status; clones detected only in treatment (“treatment-new”) or only in PBS (“PBS-new”) are explicitly annotated. All treatments generate treatment-specific clonotypes relative to PBS. (**E**) Counts of clonotypes significantly expanded versus PBS for each treatment. Across both CD4^+^ and CD8^+^ compartments, the magnitude ranks CR108 > combination > cisplatin. (**F**) Head-to-head comparison of CR108 versus cisplatin + CR108, showing that CR108 induces more “new” clonotypes than the combination in both CD4^+^ and CD8^+^ lineages. (**G**) Volcano plot for combination versus CR108 in CD4^+^ T cells, revealing a large cohort of significantly up-regulated clonotypes under the combination, consistent with selective amplification of key CD4^+^ responses that may contribute to superior antitumor efficacy.

To link chemotherapy-released antigen cargo and CR108-driven innate sensing to adaptive responses in vivo, we performed bulk TCRβ sequencing on CD4^+^ and CD8^+^ T cells isolated from tumor-draining lymph nodes (dLNs) in the 4T1 tumor model (PBS, cisplatin, CR108, or the combination). CD8^+^ repertoires displayed unimodal CDR3 length profiles with overlapping peaks across all treatments; statistical comparison revealed no significant shifts among groups, indicating that treatment did not alter gross junctional architecture over the sampling window (Figure 7A). Cisplatin monotherapy reduced the Inverse Simpson index in CD8^+^ T cells (*p* < 0.01), consistent with oligoclonal expansion of a few dominant clones, whereas Shannon entropy remained unchanged, indicating preservation of long-tail diversity. In contrast, CR108 increased the Inverse Simpson index in CD4^+^ T cells (*p* < 0.05) with a similar trend in CD8^+^, consistent with recruitment of additional clonotypes into higher-abundance ranks. Notably, the cisplatin + CR108 combo treatment restored the Inverse Simpson index to PBS-like levels despite inducing the superior tumor rejection (compare Figure 1 and Figure 7B). V–J pairing frequencies in CD8^+^ and CD4^+^ repertoires were highly similar among treatments with no groupwise drift (Figure 7C), indicating that variable gene usage and recombination structure were stable.

To resolve how these abundance changes arise, we quantified treatment-emergent (“new”) clonotypes by pairwise, depth-accounted comparisons to PBS repertoires. In scatter plots of clone-wise abundance (treatment vs. PBS), points present in treatment but not detected in PBS defined the “new-clone” set (Figure 7D). Both lineages exhibited clear emergence of treatment-specific clonotypes relative to PBS. Across CD4^+^ and CD8^+^ compartments, CR108 yielded the largest number of new clonotypes; the combination was intermediate, and cisplatin yielded the fewest (Figure 7E). A direct, head-to-head comparison between the combo treatment and CR108 (Figure 7F) reinforced this pattern: in both CD4^+^ and CD8^+^ compartments, CR108 induced a greater number of “new” clonotypes than the combination, suggesting that co-administration of cisplatin limits the recruitment of additional clones even as overall antitumor activity improves. Consistent with this, volcano analysis of clone-wise changes for combo treatment versus CR108 revealed a large set of significantly up-regulated CD4^+^ clonotypes (Figure 7G), indicating that the combination preferentially boosts the amplitude of a subset of CD4^+^ responses rather than broadly increasing breadth—an effect that may underlie its superior efficacy.

Together, these results support an antigen-centric, two-component architecture of the response without structural shifts in TCR organization: cisplatin exposure concentrates responses into fewer high-abundance clones (abundance focusing) while CR108 broadens clonotype recruitment (breadth gain) through innate immune activation; in the combo treatment, these forces balance such that breadth is tempered.

### 3.8. Soluble TA Generation/Export and Downstream Processing Associate with Favorable Outcome

**Figure 8:**
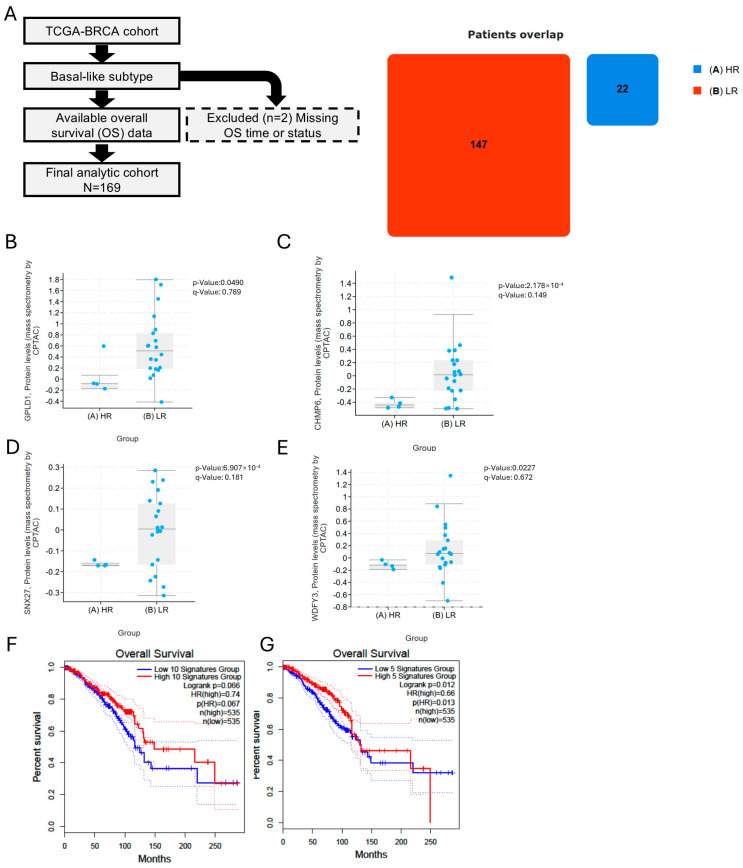
Cohort definition, soluble TAs genes, and survival analyses (**A**) Simplified CONSORT for the TCGA-BRCA basal-like analytic cohort (final *N* = 169; *n* = 2 excluded for missing/invalid OS). Right: patient-overlap counts from cBioPortal (LR = 147, HR = 22; LR indicates good prognosis, HR indicates poor prognosis). (**B**–**E**). Single-gene comparisons for GPLD1, CHMP6, SNX27, and WDFY3 between low-risk and high-risk groups (points, individual tumors; bars, median with IQR). Panel *p* values: GPLD1 *p* = 0.0490; CHMP6 *p* = 2.18 × 10^−4^; SNX27 *p* = 6.81 × 10^−4^; WDFY3 *p* = 0.0227. All four are higher in the better-prognosis arm, consistent with increased soluble TAs generation/export. (**F**,**G**). GEPIA KM OS (BRCA-basal subset) comparing High vs. Low groups defined by the 50% (median) cutoff of each gene subset: (**F**) ER loading/peptide-trimming and TAP loading subset (CALR, CANX, ERAP1, ERAP2, HSP90B1, PDIA3, TAP1, TAP2, TAPBP, TAPBPL): HRHigh = 0.74, log-rank *p* = 0.066, *n*(high) = *n*(low) = 535. (**G**) Endo/lysosomal cathepsin subset (CTSB, CTSD, CTSH, CTSS): HRHigh = 0.66, *p* = 0.012, *n*(high) = *n*(low) = 535. In both panels, High denotes better survival (HR < 1), supporting an antigen-centric framework in which soluble TA generation/export and downstream processing correlate with improved outcome.

We analyzed the TCGA-BRCA basal-like cohort in cBioPortal, dichotomizing risk at the cohort-wide KM median overall survival and excluding two cases with missing/invalid OS to yield *N* = 169 (Figure 8A; overlap: low-risk *n* = 147, high-risk *n* = 22). We then tested whether soluble tumor antigen (TA) generation/export capacity was enriched in the better-prognosis arm by comparing expression of four a priori genes—GPLD1 (GPI-anchor cleavage), CHMP6 (ESCRT-III vesicle biogenesis), SNX27 (endosomal cargo recycling), and WDFY3/ALFY (selective-autophagy routing)—and found all four higher in the low-risk group (GPLD1 *p* = 0.0490; CHMP6 *p* = 2.18 × 10^−4^; SNX27 *p* = 6.81 × 10^−4^; WDFY3 *p* = 0.0227; Figure 8B–E). Separately, restricting GEPIA to BRCA-basal and using a 50% (median) cut-point, Kaplan–Meier analyses showed improved OS for High signatures in two predefined downstream modules: ER loading/peptide trimming and TAP loading (CALR, CANX, ERAP1, ERAP2, HSP90B1, PDIA3, TAP1, TAP2, TAPBP, TAPBPL; HR_High = 0.74, log-rank *p* = 0.066; Figure 8F) and endosomal–lysosomal cathepsins (CTSB, CTSD, CTSH, CTSS; HR_High = 0.66, *p* = 0.012; Figure 8G), with *n*(High) = *n*(Low) = 535 in both comparisons. Greater capacity for soluble TA generation/export together with competent downstream processing/loading associates with superior survival in BRCA-basal, consistent with an antigen-centric mechanism of clinical benefit.

## 4. Discussion

Building on prior efficacy in 4T1, our findings show that across mechanistically diverse chemotherapies, pairing with the TLR7/8/9 agonist CR108 consistently improves antitumor immunity. This synergy was robust to heterogeneity in upstream damage responses and instead aligned with features that increase soluble TA availability, export, and competent processing/loading [25,29]. In BRCA-basal analyses, four soluble TA machinery genes (GPLD1, CHMP6, SNX27, WDFY3) were higher in the better-outcome arm, and GEPIA Kaplan–Meier in the BRCA-basal subset (50% median cutoff per gene subset) showed better OS for ER-loading/TAP and endo/lysosomal-processing signatures (HRHigh = 0.74, *p* = 0.066; HRHigh = 0.66, *p* = 0.012), supporting an antigen-centric interpretation [19,31].

While chemotherapies produced class-specific DAMP fingerprints, these patterns did not explain between-regimen differences in efficacy. Our data are more consistent with a model in which CR108-mediated DC activation amplifies pre-existing TA availability, making DAMPs permissive but not determinative in this setting [19,25].

A critical translational challenge lies in species-specific differences in TLR biology. A key translational consideration is species-specific TLR biology. While mice utilize TLR7/8/9 for nucleic acid sensing, humans exhibit dominant TLR8 expression in myeloid cells (monocytes, mDCs) and more restricted TLR9 activity primarily in B cells and pDCs. Murine pDCs robustly respond to CpG (TLR9), whereas human pDCs prioritize viral RNA sensing via TLR7. This divergence may affect TA recognition kinetics—for instance, murine pDCs respond robustly to CpG motifs (TLR9), whereas human pDCs prioritize viral RNA sensing via TLR7. To bridge this gap, future studies should employ patient-derived xenograft (PDX) models or humanized mice engrafted with human hematopoietic stem cells, which better recapitulate clinical TA-TLR interactions [32].

Although the study focused on short-term outcomes without evaluating long-term immunological memory or resistance mechanisms, which are critical for clinical durability [10,33], our prior work on cisplatin + CR108 in the 4T1 model demonstrated durable immunological memory, in which mice rejected secondary tumor challenges 60 days post-treatment [29]. This suggests that other chemo-CR108 combinations may similarly induce long-term antitumor immunity, even if their DAMP fingerprints differ. For instance, gemcitabine- and oxaliplatin-CR108 regimens elicited robust IFN-γ^+^ CD8^+^ T-cell responses (Figure 5G), a hallmark of memory T-cell priming. However, longitudinal tracking of memory T-cell subsets (e.g., central memory T cells, TCF1^+^ stem-like populations) and tertiary lymphoid structure (TLS) stability over time remains unexplored in this study. Future work should integrate re-challenge experiments across all drug combinations to validate durable protection and assess the role of metabolic programs (e.g., fatty acid oxidation) in sustaining memory responses. Additionally, profiling epigenetic regulators like TCF1 and Eomesodermin, which govern memory T-cell longevity, could mechanistically link chemotherapy-induced TAs to enduring immunity.

The TCR repertoire analysis supports an antigen-centric model of chemo–immunotherapy synergy while revealing nuanced dynamics. The reduction in Inverse Simpson diversity with cisplatin monotherapy—particularly in CD8^+^ T cells—is consistent with oligoclonal expansion of high-affinity tumor-reactive clones, likely driven by cisplatin-induced antigen exposure. This interpretation is strengthened by our ELISPOT data showing robust T-cell responses against specific TAs (e.g., Brap, Rpl13a) in the combination group. However, we acknowledge that nonantigen-specific factors like chemotherapy-induced lymphopenia or homeostatic proliferation could contribute to reduced diversity. Conversely, CR108 monotherapy expanded clonal diversity (increased Inverse Simpson index), suggesting that TLR7/8/9 agonism recruits additional T-cell specificities—potentially through enhanced DC maturation and epitope spreading (Figure 7). The combination therapy’s restoration of PBS-like diversity metrics despite profound tumor control implies a balanced remodeling: cisplatin “focuses” the repertoire on dominant tumor antigens while CR108 “broaden” coverage to subdominant epitopes, preventing excessive clonal exhaustion. The absence of structural shifts in V-J pairings or CDR3 length distributions across treatments further supports antigen-driven abundance remodeling rather than wholesale repertoire reconstruction. While inflammation-driven bystander activation remains possible, the convergence of TCR metrics with functional evidence of antigen-specific responses (Figure 3 and Figure 4) strongly favors antigen-centric selection as the dominant mechanism.

Future research should prioritize three areas: (1) leveraging single-cell omics to dissect tumor microenvironment heterogeneity post-combination therapy, (2) exploring synergies with immune checkpoint inhibitors to overcome adaptive resistance, and (3) developing biomarkers to predict optimal chemo–TLR pairings based on tumor-specific TA profiles. Additionally, clinical trials are needed to validate the safety and efficacy of CR108 combinations in human cancers, particularly in “cold” tumors resistant to monotherapy.

In conclusion, this work bridges a critical gap in chemo-immunotherapy by demonstrating that diverse chemotherapeutic mechanisms converge on CR108-amplified immunity by increasing antigen availability and enabling efficient export and processing. These insights provide a practical, antigen-centric roadmap for tailoring chemo–adjuvant strategies and advancing precision oncology.

## 5. Conclusions

Chemotherapy expands the soluble tumo antigen pool; CR108 amplified DC-mediated immunity. This synergy is conserved across tumor lineages and chemotherapeutic classes, with platinum agents inducing the broadest antigen release. TCR repertoire analysis reveals a balance between clonal focusing (chemotherapy) and diversification (adjuvant). Critically, DAMP signatures do not predict efficacy, while antigen processing machinery correlates with survival in human cancer. These findings define a unified, antigen-centric mechanism for chemo–immunotherapy synergy and offer a practical framework for clinical translation.

## Data Availability

All data reported in this paper will be shared by contacting the lead contact upon request. Any additional information required to reanalyze the data reported in this work is available from the lead contact upon request.

## Abbreviations

The following abbreviations are used in this manuscript:

APC	antigen-presenting cell
BRCA	breast invasive carcinoma (TCGA code)
CDR3	complementarity-determining region 3
CR108	TLR7/8/9 agonist used in this study
DAMP	damage-associated molecular pattern
DC	dendritic cell
dLN	tumor-draining lymph node
ELISPOT	enzyme-linked immunospot
ER	endoplasmic reticulum
ERAP1/2	endoplasmic reticulum aminopeptidase 1/2
ESCRT	endosomal sorting complexes required for transport
GEPIA	Gene Expression Profiling Interactive Analysis
GO	Gene Ontology
HSP	heat-shock protein
ICD	immunogenic cell death
ICS	intracellular cytokine staining
IFN-γ	interferon-gamma
IHC	immunohistochemistry
IQR	inter-quartile range
KEGG	Kyoto Encyclopedia of Genes and Genomes
LN	lymph node
mDC	myeloid dendritic cell
OS	overall survival
PBS	phosphate-buffered saline
PDX	patient-derived xenograft
pDC	plasmacytoid dendritic cell
q7d	once every 7 days
TAA	tumor-associated antigen
TA(s)	tumor antigen(s)
TAP	transporter associated with antigen processing
TCGA	The Cancer Genome Atlas
TCR	T-cell receptor
TCRβ	T-cell receptor beta chain
TDATTAs	tumor-derived antigenic targets
TIME	tumor immune microenvironment
TLR	Toll-like receptor
TUNEL	terminal deoxynucleotidyl transferase dUTP nick-end labeling
TRBJ	TCR beta joining gene
TRBV	TCR beta variable gene
i.v.	intravenous
s.c.	subcutaneous
